# Effect of Exogenous Ketones as an Adjunct to Low-Calorie Diet on Metabolic Markers

**DOI:** 10.3390/nu17223582

**Published:** 2025-11-16

**Authors:** Eliza J. Roeth, Genevieve Parker, Ella F. Cooper-Leavitt, Colson G. Beus, Cameron R. Braithwaite, Madeline D. Morris, Asher P. Reynolds, Ethan P. Evans, Jack H. Radford, Fischer D. Davis, Paul R. Reynolds, R. Ryley Parrish, Benjamin T. Bikman

**Affiliations:** 1 Department of Cell Biology and Physiology, Brigham Young University, Provo, UT 84602, USA; 2 Department of Mathematics, Utah Tech University, St. George, UT 84770, USA

**Keywords:** ketones, weight loss, body composition, beta-hydroxybutyrate

## Abstract

Background/Objectives: Overweight and obesity affect a majority of adults, contributing to metabolic disorders. Caloric restriction often leads to undesirable lean mass loss alongside fat reduction. This study investigated whether exogenous β-hydroxybutyrate (BHB) supplementation, as an adjunct to a hypocaloric diet, improves body composition and metabolic markers in overweight and obese adults by preferentially reducing fat mass while preserving lean mass. Methods: In this 8-week randomized, double-blind, placebo-controlled trial, 51 adults were assigned to receive either racemic BHB mineral salts or placebo (maltodextrin) twice daily, alongside modest caloric restriction. Assessments at baseline and week 8 included dual-energy X-ray absorptiometry for body composition, indirect calorimetry for resting metabolic rate (RMR), and venous blood analyses for cardiometabolic biomarkers (e.g., lipids, HOMA-IR, uric acid, liver enzymes). Results: Body mass decreased in both groups over the intervention (*p* < 0.01 within placebo and *p* < 0.001 within BHB). Within the BHB group, fat mass decreased significantly (−2 kg; *p* < 0.05 vs. baseline), body fat percentage improved (*p* < 0.01 vs. baseline), and lean-to-fat mass ratio increased (*p* < 0.05 vs. baseline); no such significant changes were observed within the placebo group. Group × time interactions were not significant for these body composition variables (*p* > 0.05). Furthermore, lean mass was largely preserved, with no declines in RMR. Within the BHB group, LDL cholesterol was reduced (*p* < 0.05 vs. baseline), while other lipids, HOMA-IR, and uric acid remained stable, with liver enzymes showing a positive change. Conclusions: Exogenous BHB supplementation may enhance the quality of diet-induced weight loss through within-group improvements in fat mass reduction and lean mass preservation, with no adverse metabolic impacts.

## 1. Introduction

Overweight and obesity continue to pose substantial public health challenges, with over two-thirds of adults in the U.S. currently classified as either overweight or obese [1]. Excess adiposity is causally linked to insulin resistance, type 2 diabetes, dyslipidemia, hypertension, cardiovascular disease, nonalcoholic fatty liver disease, and increased mortality risk [2]. Consequently, interventions that reduce total and visceral fat mass while preserving lean mass are of paramount importance in both preventive and therapeutic contexts. Obesity is now a global, rapidly rising burden. In 2022 more than one billion people were living with obesity, including ~880 million adults and ~159 million youths, with prevalence more than doubling in adults since 1990 and quadrupling in youth [3].

The persistence of obesity reflects both biological and environmental barriers to sustained weight reduction. Weight loss typically triggers metabolic adaptations that favor weight regain, including reductions in resting energy expenditure and increased hunger drive [4]. In addition, highly processed and calorie-dense foods are ubiquitous in modern food environments, making long-term adherence to healthy eating patterns difficult [5]. As a result, lifestyle interventions often achieve modest and transient success, highlighting the need for complementary strategies to improve body composition and metabolic health.

Most conventional weight-loss strategies rest on the principle of a sustained energy deficit (i.e., energy intake < energy expenditure). However, weight loss induced by caloric restriction alone tends to produce heterogeneous outcomes: in particular, a substantial proportion of the lost mass is often lean tissue (fat-free mass), which includes skeletal muscle, bone, other organs, and water. Indeed, in many well-controlled weight-loss protocols, a substantial portion of mass lost is fat-free tissue, sometimes approaching 20–30% of total weight lost in aggressive interventions [6]. This loss of lean mass during weight loss is not benign; reductions in metabolically active tissue contribute to declines in resting metabolic rate, impairments in physical function, and increased susceptibility to weight regain (a phenomenon sometimes termed “metabolic adaptation”) [7].

The challenge is thus twofold: achieve a sufficiently large fat mass reduction, while minimizing lean mass loss. Macronutrient composition (especially protein dose and carbohydrate/fat balance) is one modifiable lever that may influence the balance of fat vs. lean tissue loss. Under carbohydrate restriction, the liver shifts substrate metabolism toward ketogenesis, producing β-hydroxybutyrate (BHB) and acetoacetate, which can be oxidized by multiple tissues (including brain, heart, kidney, and skeletal muscle) when glucose consumption is restricted. Some evidence suggests that ketogenic diets might reduce the proportion of lean mass lost during weight reduction—though findings are inconsistent, and long-term adherence is a challenge for any dietary change [8].

One practical limitation of ketogenic diets is that many individuals find it difficult to sustain strict carbohydrate restriction for extended periods, especially in free-living settings. This has catalyzed interest in exogenous ketone supplementation as a strategy to elevate circulating ketone levels independent of macronutrient restriction. Exogenous ketone compounds include ketone esters, ketone mineral salts, and formulations combining ketones with medium-chain triglycerides (MCTs) [9,10]. Oral consumption of these compounds has been shown to acutely raise plasma β-hydroxybutyrate concentrations—sometimes within 30–60 min—without the need for prolonged carbohydrate restriction [9,11]. However, exogenous ketones that emphasize BHB over alcohol precursors, such as 1,3-butanediol, spare any obvious liver pathologies [12].

Ketones have long been considered “muscle sparing”. Classically, this view, espoused by George Cahill, rested on nutrient metabolism—with availability of ketones, muscle amino acids avoid proteolysis and subsequent hepatic-driven gluconeogenesis [13,14,15]. More recent evidence adds mechanistic insight to this. BHB may inhibit muscle catabolism via modulation of mTOR signaling [16,17], as well as possibly increasing anti-catabolic regulators (e.g., IGF1, growth hormone) [18].

Despite these observations, the degree to which exogenous BHB is capable of mitigating lean mass loss in calorie restriction remains unknown. The present study was designed to address this gap. We sought to examine the effects exogenous BHB, implemented concurrently with a hypocaloric diet, on total body weight, fat mass, lean mass, lean–fat ratio, resting metabolic rate, and other key cardiometabolic biomarkers in overweight and obese adults.

## 2. Methods

### 2.1. Study Design

This study was conducted over an eight-week period using a randomized, double-blind, placebo-controlled, parallel-groups design. Participants completed three visits: an initial screening, a baseline assessment, and a final assessment at week 8. Screening procedures included written informed consent, medical history, physical examination, and routine clinical blood work. At baseline and week 8, participants completed assessments of body composition, resting metabolic rate, venous blood collection, and anthropometrics. Prior to each testing visit, participants were instructed to replicate dietary intake from the previous 24 h, abstain from exercise for 48 h, and refrain from alcohol and caffeine consumption for 24 h. This analysis focuses on the placebo and one BHB formulation (racemic BHB mineral salts) from a larger multi-arm trial examining multiple ketone types; other formulations are reported separately. Participants were also instructed to perform 30 min of walking at least 3 days per week, consistent with the parent study protocol. All study procedures were approved by the institutional review board (Protocol: KETAD-001-2018; approval date: 25 January 2019), and the trial was conducted in accordance with the Declaration of Helsinki.

### 2.2. Participants

A total of 51 adults (male, *n* = 20; female, *n* = 31) between the ages of 18 and 46 years were enrolled (34.6 ± 6.7 years, 171.2 ± 10.3 cm, 92.6 ± 14.9 kg, BMI 31.4 ± 2.9 kg/m^2^). Subjects were randomly assigned to either the placebo (*n* = 27) or treatment group (*n* = 24). The target sample size (*n* = 51; 27 placebo, 24 BHB) was determined based on feasibility considerations and sample sizes used in comparable nutrition trials examining body composition responses to ketone supplementation. Post hoc power analysis indicated that this sample provided approximately 80% power (α = 0.05) to detect a between-group difference of 1.5 kg in fat mass. Inclusion criteria required participants to have a BMI between 27 and 35 kg/m^2^, be weight-stable (±2.3 kg in the previous 30 days), and classified as normotensive (systolic < 140 mmHg, diastolic < 90 mmHg, resting heart rate < 90 beats/min). Exclusion criteria included pregnancy, nursing, as well as any history of metabolic disease (e.g., diabetes, thyroid disorders), cardiovascular disease, hepatic or renal dysfunction, autoimmune or neurological conditions. Individuals taking dietary supplements or medications known to alter body weight, metabolism, or hormone levels within four weeks of study initiation were excluded. During weekly phone calls, the frequency and intensity of local and systemic non-serious and serious adverse events (AEs) were recorded by study team members.

### 2.3. Dietary Intake and Control

During the initial screening visit, participants were asked to complete a 24 h dietary recall to assess general habits, food restrictions, diet composition and intake. All subjects were placed on a “Zone” type diet (~40% carbohydrates, 30% protein, 30% fat) that provided approximately 500 kcals per day less than their estimated energy requirements calculated with the Mifflin St. Jeor equation. This composition was selected to (1) avoid endogenous nutritional ketosis by maintaining moderate carbohydrate intake, thereby isolating the effects of exogenous BHB; (2) provide relatively higher protein during energy restriction to help preserve lean mass; and (3) promote adherence and satiety with moderate fat without initiating a high-fat regimen that might independently influence lipid metabolism. Upon the initial dietary assignment, the research dietitian met with each subject to explain the proper procedures for recording dietary intake and provide examples of the types of foods they could consume while also providing instruction to facilitate understanding and general compliance to the diet. Throughout the eight-week study, three-day dietary records (including two weekdays and one weekend day) were completed during weeks 0, 4, and 8 to assess general compliance to the protocol and to further assess if dietary changes occurred. Dietary records were recorded with the MyFitnessPal application to obtain average daily energy and macronutrient intake. Copies of food records were made and provided to each study participant to allow them to standardize their dietary and fluid intake prior to each laboratory visit. In addition, weekly contact/communication occurred between study participants and team members regarding dietary compliance.

### 2.4. Anthropometrics and Resting Metabolic Rate

Height was measured using a wall-mounted stadiometer, and body mass was measured with a calibrated digital scale (Seca 767™, Hamburg, Germany). Resting metabolic rate (RMR) was assessed using indirect calorimetry (ParvoMedics TrueOne^®^ 2400, Sandy, UT, USA). Participants reported to the laboratory following a 10 h overnight fast and were tested in the morning in a thermoneutral, dimly lit environment. After calibration of gas and volume analyzers, participants rested in a semi-reclined position while wearing a headgear system with an oro-nasal mask (Hans Rudolph 7450, Hans Rudolph, Inc., Shawnee, KS, USA). Expired gases were analyzed continuously, and data were visually inspected to identify a steady-state 5 min window of minimal variability in VO_2_ and VCO_2_. This value was used to calculate RMR, expressed relative to body mass (kcal·kg^−1^·day^−1^).

### 2.5. Body Composition

Body composition was assessed by dual-energy X-ray absorptiometry (DEXA; GE Lunar DPX Pro, Madison, WI, USA) at baseline and week 8. Outcomes included fat mass, lean mass, percent body fat, and the lean–fat mass ratio. Scans were conducted by the same trained technician and analyzed using enCORE software (version 13.31). Standardized positioning protocols and anatomical landmarks were applied, and participants remained motionless for approximately 10 min during each scan. Daily calibration with a phantom block was conducted to ensure quality control. The reliability of repeated measurements with this device in our laboratory has previously demonstrated intraclass correlation coefficients >0.98 for lean mass, fat mass, and bone mineral content.

### 2.6. Blood Collection and Analyses

Venous blood samples were collected after a 10 h fast at baseline and week 8. Whole blood was collected into EDTA tubes and serum into separation tubes, then centrifuged at 3200 rpm for 15 min at room temperature (Horizon Mini E, Drucker Diagnostics, Port Matilda, PA, USA). Samples were analyzed by a central laboratory (LabCorp, Dublin, OH, USA). Plasma insulin and glucose were used to calculate homeostatic model assessment for insulin resistance (HOMA-IR) using the standard formula: HOMA-IR = [glucose (mg/dL) × insulin (µU/mL)]/405. The laboratory panel included fasting glucose, insulin, total cholesterol, HDL cholesterol, LDL cholesterol, triglycerides, uric acid, alanine aminotransferase (ALT), aspartate aminotransferase (AST), and alkaline phosphatase (ALP). All analyses were conducted using standard enzymatic or immunoassay methods in a CLIA-certified laboratory.

### 2.7. Supplementation

Participants were stratified by sex and BMI, then randomized to one of two groups in a double-blind manner: (1) placebo (maltodextrin), or (2) racemic β-hydroxybutyrate (BHB) mineral salts (BHB). Supplements were provided as powders, matched for taste and appearance, and packaged in coded containers, with each containing 5g of their respective ingredient. This twice-daily 5 g racemic BHB-salt regimen was chosen to elicit modest, physiologic ketonemia while preserving tolerability and adherence, consistent with human data showing ketone salts can raise D-βHB to ~1.0 mM at higher single doses and to lower, yet physiologically relevant, levels at smaller doses [19]. Participants consumed one serving in the morning and one in the late afternoon, each dissolved in 240 mL of water, for eight weeks. Compliance was monitored through daily logs and weekly check-ins.

### 2.8. Statistical Analyses

Data were analyzed using SPSS version 23 (IBM, Armonk, NY, USA) and graphed via GraphPad Prism 10 (Boston, MA, USA). Group differences at baseline were evaluated by independent *t*-tests. Intervention effects were examined using two-way ANOVA (group × time) with repeated measures on time. Significant interactions were followed by post hoc comparisons using change scores (Δ = week 8 − baseline). Within-group changes were assessed by paired *t*-tests. Results are presented as mean ± standard deviation, with significance set at *p* < 0.05.

## 3. Results

### 3.1. Cardiometabolic Markers

**Figure 1:**
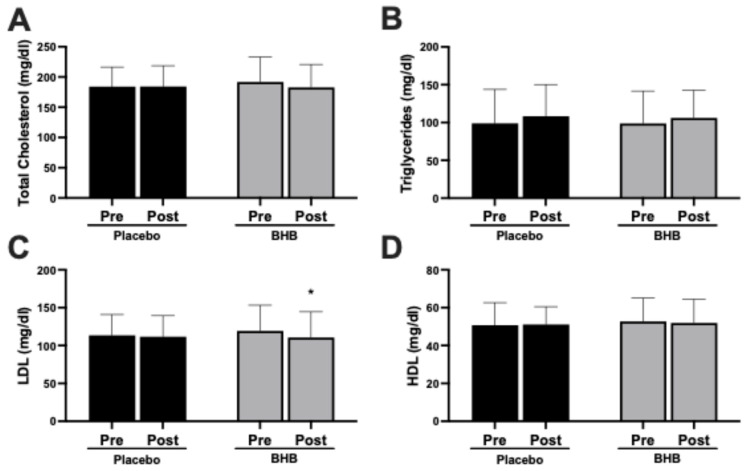
Effects of exogenous ketone supplementation on blood lipids. (**A**) Total cholesterol (mg/dL) in pre- and post-intervention in placebo (black) and BHB-supplemented (grey) groups. (**B**) Triglycerides (mg/dL) in pre- and post-intervention in placebo (black) and BHB-supplemented (grey) groups. (**C**) LDL cholesterol (mg/dL) in pre- and post-intervention in placebo (black) and BHB-supplemented (grey) groups. (**D**) HDL cholesterol (mg/dL) in pre- and post-intervention in placebo (black) and BHB-supplemented (grey) groups. Data are presented as mean ± SD, 27 subjects in the placebo group and 24 in the BHB group. * *p* < 0.05 vs. pre-intervention.

Baseline demographic data are shown in Table A1 in Appendix A and adverse events reporting are shown Table A2. Blood lipids were analyzed across the 8-week intervention. No changes were observed in total cholesterol (Figure 1A), triglycerides (Figure 1B), or high-density lipoprotein (HDL) cholesterol (Figure 1D). However, low-density lipoprotein (LDL) cholesterol levels remained stable in the placebo group but decreased significantly in the BHB-supplemented group (*p* < 0.05 within-group; Figure 1C). These data are also found in Table A4.

### 3.2. Metabolic and Renal Markers

**Figure 2:**
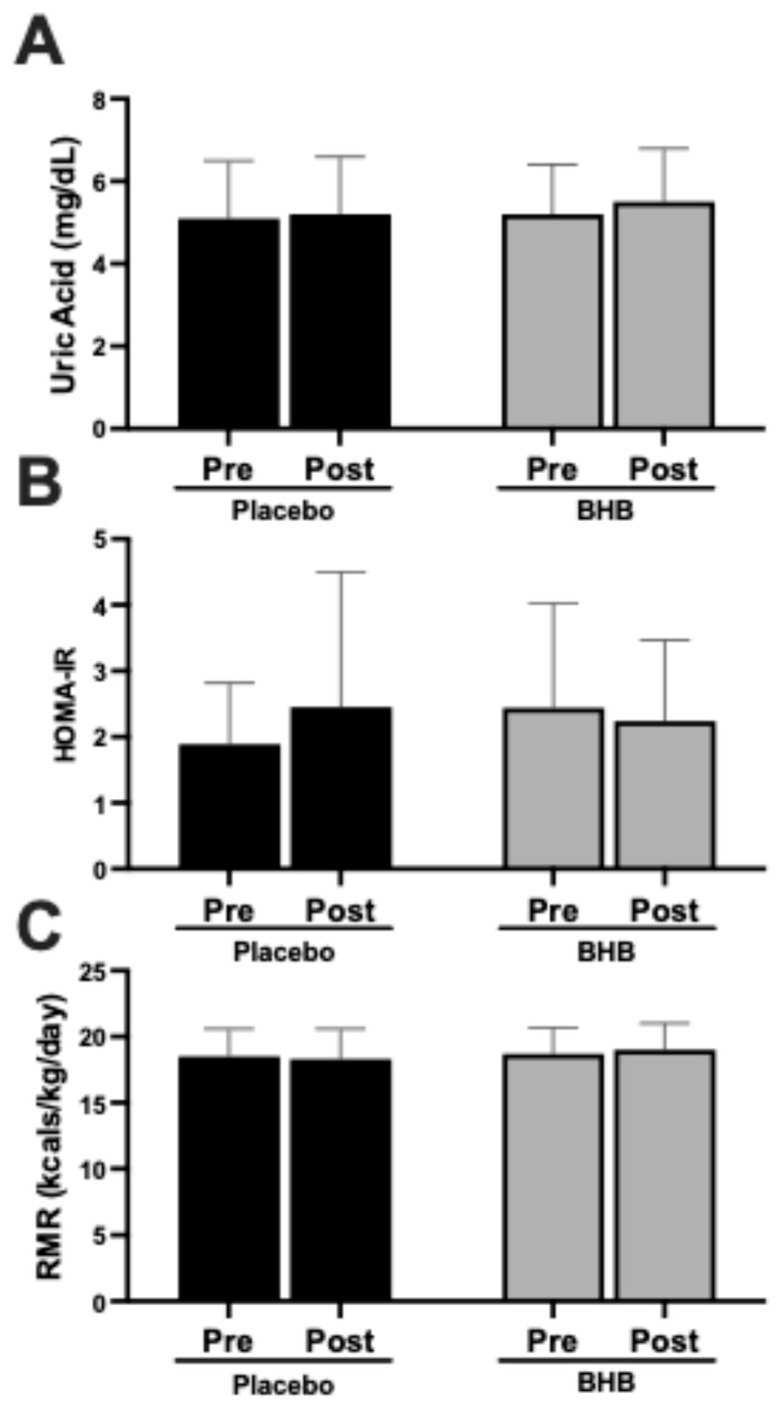
Effects of exogenous ketone supplementation on uric acid, insulin resistance, and resting metabolic rate. (**A**) Uric acid (mg/dL) pre- and post-intervention in placebo (black) and BHB-supplemented (grey) groups. (**B**) HOMA-IR pre- and post-intervention in placebo (black) and BHB-supplemented (grey) groups. (**C**) Resting metabolic rate (kcals/kg/day) pre- and post-intervention in placebo (black) and BHB-supplemented (grey) groups. Data are presented as mean ± SD, 27 subjects in the placebo group and 24 in the BHB group. No significant differences were observed.

Uric acid levels showed no significant alterations from pre- to post-intervention in either group (Figure 2A). Insulin resistance, as measured by HOMA-IR, remained unchanged across all participants (Figure 2B), though a trend of increased levels were noted in the placebo group. Resting metabolic rate (RMR) was preserved in both the placebo and BHB-supplemented groups, with no evidence of declines indicative of metabolic adaptation (Figure 2C). These null findings further underscore the absence of adverse effects on metabolic or renal health. These data are also found in Table A4.

### 3.3. Body Mass and Body Fat Percentage

**Figure 3:**
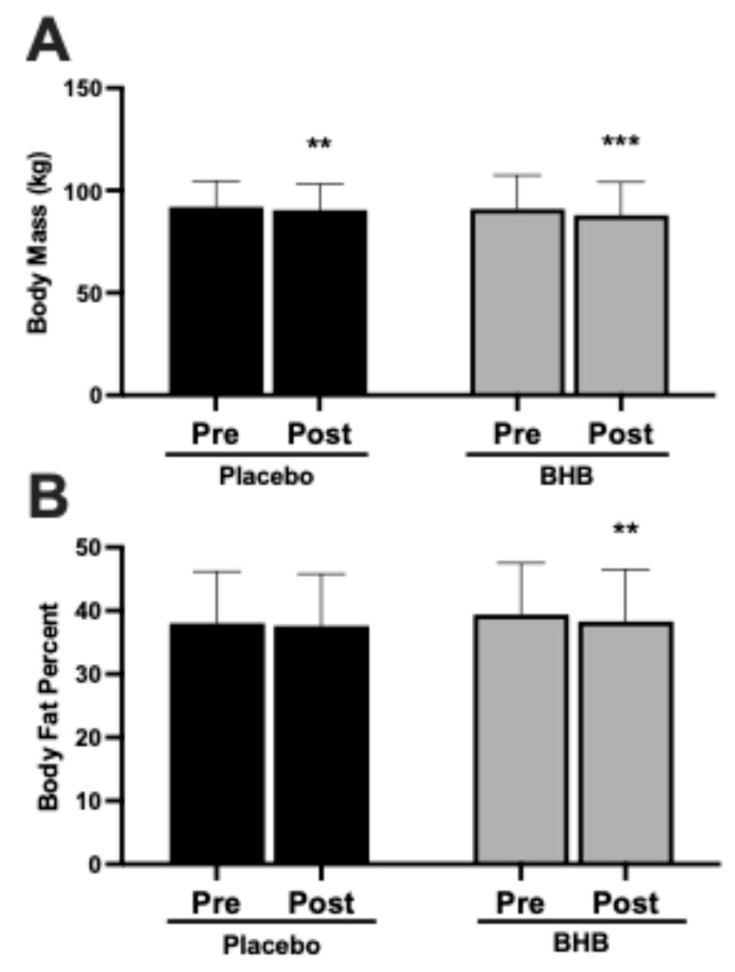
Effects of exogenous ketone supplementation on body mass and body fat percentage. (**A**) Body mass (kg) pre- and post-intervention in placebo (black) and BHB-supplemented (grey) groups. (**B**) Body fat percent pre- and post-intervention in placebo (black) and BHB-supplemented (grey) groups. Data are presented as mean ± SD, 27 subjects in the placebo group and 24 in the BHB group. ** *p* < 0.01, ***p < 0.001 vs. pre-intervention within group as indicated.

Body mass decreased over the intervention in both groups (*p* < 0.01 within placebo and *p* < 0.001 within BHB; Figure 3A), with mean changes of approximately −1 kg in placebo and −3 kg in the BHB-supplemented group. Body fat percentage decreased significantly within the BHB-supplemented group (*p* < 0.01 vs. baseline; Figure 3B) but not within the placebo group. The group × time interaction was not significant for body fat percentage (*p* > 0.05; Table A3).

### 3.4. Fat Mass, Lean Mass, and Lean-to-Fat Mass Ratio

**Figure 4:**
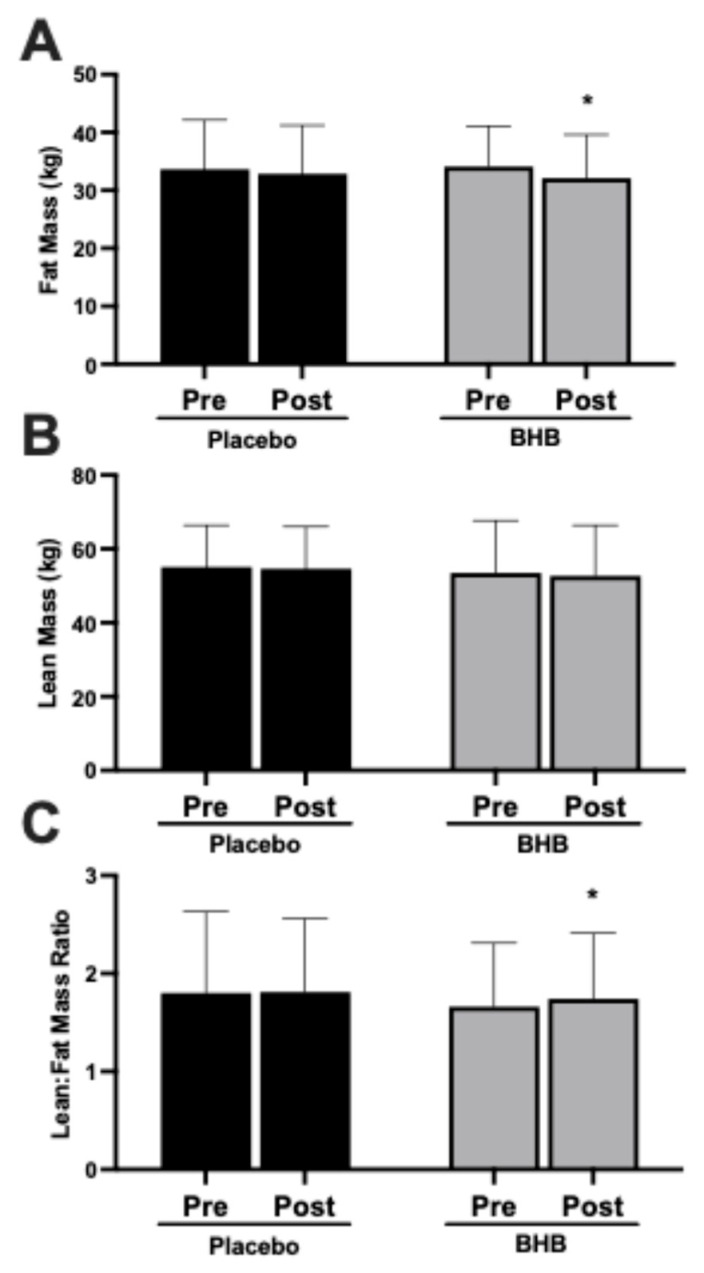
Effects of exogenous ketone supplementation on body composition. (**A**) Fat mass (kg) pre- and post-intervention in placebo (black) and BHB-supplemented (grey) groups. (**B**) Lean mass (kg) pre- and post-intervention in placebo (black) and BHB-supplemented (grey) groups. (**C**) Lean-to-fat mass ratio pre- and post-intervention in placebo (black) and BHB-supplemented (grey) groups. Data are presented as mean ± SD, 27 subjects in the placebo group and 24 in the BHB group. * *p* < 0.05 vs. pre-intervention within group as indicated.

Fat mass decreased significantly within the BHB-supplemented group (*p* < 0.05 vs. baseline; Figure 4A) but not within the placebo group. The group × time interaction was not significant for fat mass (*p* > 0.05; Table A3). Lean mass was largely preserved in both groups, with no significant changes from baseline (Figure 4B). The lean-to-fat mass ratio improved significantly within the BHB-supplemented group (*p* < 0.05 vs. baseline; Figure 4C) but showed no significant change within the placebo group. The group × time interaction was not statistically significant for lean-to-fat mass ratio (*p* = 0.165; Table A3).

### 3.5. Liver Enzymes

**Figure 5:**
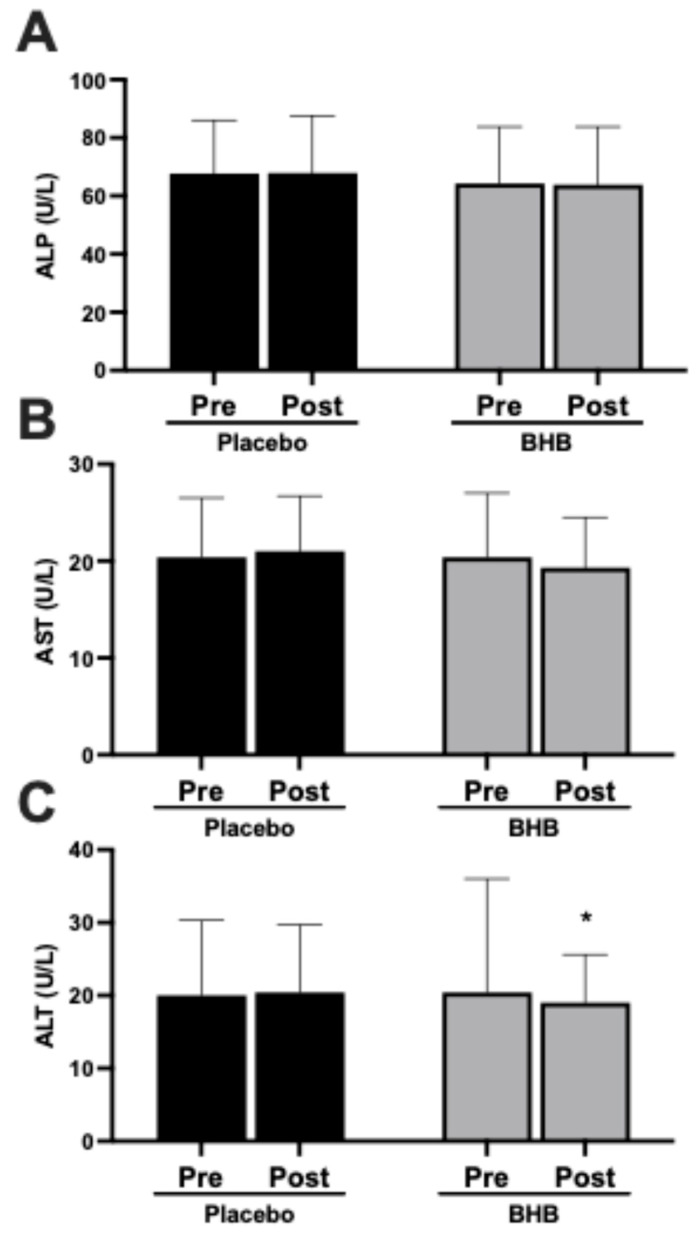
Effects of exogenous ketone supplementation on liver enzymes. (**A**) Alkaline phosphatase (ALP; U/L) pre- and post-intervention in placebo (black) and ketone-supplemented (grey) groups. (**B**) Aspartate aminotransferase (AST; U/L) pre- and post-intervention in placebo (black) and ketone-supplemented (grey) groups. (**C**) Alanine aminotransferase (ALT; U/L) pre- and post-intervention in placebo (black) and ketone-supplemented (grey) groups. Data are presented as mean ± SD, 27 subjects in the placebo group and 24 in the BHB group. * *p* < 0.05 vs. pre-intervention.

In light of recent evidence suggesting a harm from exogenous ketone precursors, we aimed to determine liver health in the current study. Alkaline phosphatase (ALP) and aspartate aminotransferase (AST) levels remained stable across the intervention in both groups (Figure 5A,B). Alanine aminotransferase (ALT) showed a slight but significant decrease in the BHB-supplemented group (*p* < 0.05 within-group; Figure 5C). These data are also found in Table A4.

## 4. Discussion

### 4.1. Summary of Main Findings

The primary finding of this study is that, within the BHB-supplemented group paired with modest caloric restriction, participants exhibited modest but statistically significant improvements in body composition compared to baseline—including reductions in fat mass and body fat percentage, with proportional preservation of lean tissue leading to a more favorable lean-to-fat mass ratio. In contrast, no such significant changes were observed within the placebo group for these measures. While group × time interactions were not statistically significant (e.g., *p* > 0.05 for fat mass and lean–fat ratio), the within-group effects in the BHB arm suggest potential benefits warranting further investigation.

This differential outcome is particularly relevant given that lean mass loss commonly accompanies caloric restriction, often comprising up to 20–30% of total weight lost [20,21,22]. Preservation of metabolically active tissue is critical for maintaining resting metabolic rate (RMR), physical function, and long-term weight maintenance. The current results align with prior evidence that elevated BHB levels can reduce leucine oxidation and support muscle protein synthesis [15]. Ketones may act both as a readily oxidizable fuel for skeletal muscle and as signaling molecules that influence muscle preservation by modulating pathways involved in protein turnover and inflammation [16].

### 4.2. Cardiometabolic Outcomes and Metabolic Stability

Secondary outcomes further support the metabolic safety and neutrality of exogenous ketones during caloric restriction. Across all groups, no adverse changes were observed in insulin resistance (HOMA-IR), uric acid, or RMR (Figure 1 and Figure 2). The preservation of RMR, in particular, is noteworthy, as lean tissue loss is a key driver of metabolic slowdown during diet-induced weight loss. Maintenance of metabolic rate may help support continued weight loss and reduce the likelihood of rebound weight gain.

Notably, participants receiving ketones experienced a significant reduction in LDL cholesterol (Figure 1C), which is somewhat uncommon during periods of fat loss. Caloric restriction and lipid mobilization commonly elevate circulating cholesterol as adipose stores are released into the bloodstream [23]. The observed LDL decline may reflect enhanced hepatic clearance or shifts in cholesterol transport and metabolism induced by BHB. These findings merit further investigation, but they suggest that exogenous ketones do not compromise, and may even modestly benefit, lipid profiles during energy deficits.

### 4.3. Mechanistic Insights: Potential Positive Effects of BHB

Beyond serving as an alternative oxidative substrate, β-hydroxybutyrate exerts pleiotropic cellular effects that may underlie the favorable body-composition and metabolic outcomes observed here. BHB functions as a signaling metabolite that influences gene expression and cellular redox state. It acts as an endogenous inhibitor of class I histone deacetylases (HDACs), thereby promoting expression of oxidative stress–resistance genes such as FOXO3A, SOD2, and CAT [24]. This mechanism may contribute to reduced inflammation and improved mitochondrial efficiency during caloric restriction. BHB also activates the G-protein–coupled receptor HCAR2 (GPR109A) on adipocytes and immune cells, leading to inhibition of lipolysis, enhanced adiponectin secretion, and suppression of pro-inflammatory cytokines such as TNF-α and IL-1β [25].

In skeletal muscle, BHB provides an efficient energy source that spares glucose and branched-chain amino acids from oxidation, thereby preserving muscle protein stores [26]. BHB has additionally been shown to attenuate activation of the NLRP3 inflammasome, which is implicated in metabolic inflammation and insulin resistance [25]. Through these combined effects—epigenetic modulation, receptor signaling, anti-inflammatory action, and substrate sparing—exogenous BHB supplementation may enhance metabolic resilience, maintain lean tissue, and promote healthier lipid handling during energy restriction. These mechanistic pathways likely explain the observed improvements in body composition and lipid profile in the present study.

### 4.4. Relevance to GLP-1 Agonist Therapies

The lean mass-sparing effects of exogenous ketones hold strong translational potential, particularly in the context of pharmacological weight-loss therapies such as glucagon-like peptide-1 (GLP-1) receptor agonists (e.g., semaglutide). While these agents can produce substantial weight loss, body composition analyses have revealed disproportionately high lean mass loss. For example, in the STEP 1 trial, once-weekly semaglutide at 2.4 mg led to a 14.9% reduction in total body weight over 68 weeks [27]. However, follow-up analyses reported a 9.7% decrease in lean mass, comprising up to 40% of the total weight lost, while fat mass decreased by 19.3% [28].

This degree of lean tissue loss—generally exceeding that seen with caloric restriction alone—raises concerns about impaired functional capacity, increased frailty risk, and difficulty sustaining weight loss over time. Our findings suggest that BHB supplementation may shift the balance toward fat oxidation while preserving muscle. While pharmacological efforts are in pursuit of combined therapies to mitigate lean mass loss with GLP-1-based therapies, future studies should evaluate the benefit of including exogenous ketones in this context to determine whether they can further enhance body composition outcomes with fewer side effects.

### 4.5. Liver Health and Formulation Safety

Recent preclinical studies have highlighted critical differences between ketone formulations in their effects on liver health. In a recently published preclinical report [12], chronic supplementation with BHB salts, like that used in the current study, preserved hepatic structure, reduced inflammatory cytokines (e.g., TNF-α), and minimized steatosis. In contrast, ketone esters and precursors such as 1,3-butanediol were associated with increased lipid droplet accumulation, vascular congestion, elevated ALT and AST levels, and greater inflammatory stress. Specifically, BHB salts showed the lowest hepatic fat infiltration and arginase induction, indicating minimal metabolic burden, while 1,3-butanediol and ester forms produced hepatocellular ballooning and immune infiltration.

Our results revealed that liver enzyme profiles remained within normal ranges across all groups, with no significant changes in AST or ALP and, in fact, a modest reduction in ALT in the BHB groups (Figure 5). These findings support the hepatic safety of the tested BHB salt formulations over an 8-week period.

These divergent outcomes underscore the importance of formulation choice. For long-term or clinical use—particularly in individuals with nonalcoholic fatty liver disease (NAFLD) or other hepatic concerns—BHB should be prioritized over less stable or more metabolically taxing precursors.

### 4.6. Strengths and Limitations

This study benefits from several methodological strengths: a randomized, double-blind, placebo-controlled design; a relatively large sample size for a nutrition trial; and the use of dual-energy X-ray absorptiometry (DEXA) for accurate body composition assessment. The free-living model, incorporating modest caloric restriction and light physical activity, further enhances ecological validity and mirrors realistic lifestyle conditions under which many individuals attempt weight loss. However, limitations include the absence of plasma ketone measurements, which precludes confirmation of ketonemia levels or participant compliance. Because circulating or urinary BHB concentrations were not directly measured, the present results should be interpreted as associative, and causality between exogenous BHB ingestion and body-composition changes cannot be definitively established. Nevertheless, previous studies have shown that the dose of BHB used in this study is sufficient to induce ketonemia [29,30,31]. The relatively short intervention duration may not have captured longer-term trends or modest between-group effects in lean mass. Finally, dietary intake was assessed by self-reported food records, which are subject to recall bias and reporting inaccuracies that may have introduced measurement error in estimating actual macronutrient intake. An additional limitation is that this report represents a subset analysis from a larger multi-arm trial; while randomization was preserved, power for between-group comparisons may be reduced.

### 4.7. Future Directions

Given the increasing concern around lean mass loss in both traditional and pharmaceutical weight-loss interventions, further investigation into the protective effects of exogenous ketones is warranted. Future studies should be longer in duration and include direct assessments of circulating ketone levels to verify compliance and characterize dose–response relationships. Functional outcomes such as strength, endurance, balance, and mobility should be measured alongside body composition to evaluate the preservation of muscle quality, not just quantity.

There is also a compelling need to assess the impact of exogenous ketones in combination with GLP-1 receptor agonists in both younger and older adults, including postmenopausal women and individuals at risk for sarcopenia. Stratified analyses by age, sex, and baseline metabolic status could help determine whether certain populations benefit more from ketone co-administration.

## 5. Conclusions

In conclusion, eight weeks of caloric restriction produced weight and fat loss in both groups. Within the exogenous ketone supplementation group, there was a favorable, albeit modest, shift in body composition compared to baseline, with reductions in fat mass and preservation of lean mass leading to improved lean–fat ratios; no such significant changes were observed within the placebo group. Group × time interactions were not significant for key body composition variables. These within-group findings suggest ketones may enhance the quality of weight loss, potentially addressing one of the key limitations of current diet and drug strategies, though larger studies are needed to confirm effects relative to placebo.

## Data Availability

The original contributions presented in this study are included in the article. Further inquiries can be directed to the corresponding author.

## Appendix A

**Table A1:** Baseline characteristics.

	Group	Mean ± SD	*p*-Value *
Age (years)	PLA QBHB	34.9 ± 6.1 33.9 ± 35.9	0.57
Height (centimeters)	PLA QBHB	172.2 ± 9.5 168.9 ± 12.6	0.81
Body Mass (kilograms)	PLA QBHB	91.7 ± 12.5 90.8 ± 16.5	0.57
Body Mass Index (kg/m^2^)	PLA QBHB	30.8 ± 2.7 31.7 ± 2.9	0.64
DEXA % Fat	PLA QBHB	38.0 ± 8.2 39.4 ± 8.1	0.90

* *p* < 0.05.

**Table A2:** Adverse Events Reporting.

	Allocated Subjects (*n* = 51)	
	Placebo (*n* = 27)	BHB (*n* = 24)
Severity		
Mild	2	5
Moderate		
Severe		
Relationship to Study Treatment		
Not related		1
Possible	2	2
Definite		
Relationship to Test Article		
Not related		
Possible	2	2
Definite		
Body System and AEs		
Gastrointestinal		
abdominal distension; bloating	2	
nonspecific; diarrhea		
GI inflammatory disorder; abdominal pain		
motility; constipation		1
motility; defecation frequency decreased		
motility; frequent bowel movements		2
nausea		
oral dryness & salivary; dry mouth		
Hematology Investigations		
liver function investigation; increased AST & ALT		
Immune		
Hypersensitivity; urticaria (hives)		
Nervous System		
headache		
Renal & Urinary		
dysuria; micturition burning		
Surgical & Medical Procedures		
venipuncture; diaphoresis		
Total Number of AE Experienced During Study	2	3
Total Number of Subjects Experiencing AE: *n*	2	3

**Table A3:** Anthropometric and Body Composition Outcomes.

Variables	*n*	Baseline (Week 0)	Post (Week 8)	Delta	Within (*p*)	Group × Time (*p*)
Body Mass (kg)						
PLA	27	91.9 ± 12.8	90.4 ± 12.8	−1.51 ± 2.61 †	0.006	0.09
BHB	24	90.9 ± 16.5	87.9 ± 16.2	−3.05 ± 2.53 †	<0.001	
Body Mass Index (kg/m^2^)						
PLA	27	30.9 ± 2.8	30.4 ± 2.6	−0.52 ± 0.88	0.005	0.08
BHB	24	31.7 ± 2.8	30.7 ± 2.8	−1.07 ± 0.90	<0.001	
Waist Circumference (cm)						
PLA	27	98.2 ± 9.3	96.1 ± 9.4	−2.1 ± 6.3	0.09	0.69
BHB	24	96.4 ± 11.1	94.9 ± 10.9	−1.5 ± 5.9	0.23	
Hip Circumference (cm)						
PLA	27	110.6 ± 7.5	109.8 ± 7.7	−0.8 ± 4.0 †	0.29	0.05
BHB	24	110.5 ± 7.2	105.9 ± 5.7	−4.6 ± 5.4 †	<0.001	
DEXA Fat Mass (kg)						
PLA	27	33.6 ± 8.5	32.8 ± 8.4	−0.82 ± 2.39 †	0.09	0.22
BHB	24	34.1 ± 7.0	32.1 ± 7.5	−1.97 ± 2.32 ††	<0.001	
DEXA Lean Mass (kg)						
PLA	27	55.1 ± 11.3	54.6 ± 11.4	−0.45 ± 1.95	0.25	0.58
BHB	24	53.5 ± 14.2	52.7 ± 13.7	−0.85 ± 2.09	0.06	
DEXA Percent Fat (%)						
PLA	27	38.0 ± 8.2	37.6 ± 8.2	−0.38 ± 2.07	0.35	0.54
BHB	24	39.4 ± 8.1	38.3 ± 8.2	−1.11 ± 2.03	0.01	
DEXA Lean–Fat Ratio						
PLA	27	1.80 ± 0.83	1.81 ± 0.75	0.013 ± 0.20 †	0.75	0.43
BHB	24	1.66 ± 0.65	1.74 ± 0.67	0.09 ± 0.19	0.04	

The between-group *p*-value was assessing the group x time interaction between week 0 and week 8. † Different than group BHB, *p* < 0.05. ††, *p* < 0.01.

**Table A4:** Laboratory Parameters.

Variables	*n*	Baseline (Week 0)	Post (Week 8)	Within (*p*)	Delta	Between-Group *p*-Value *
Glucose (grams/dL)						
PLA	27	90.2 ± 5.9	89.0 ± 9.1	−1.22 ± 9.86	0.53	0.09
BHB	24	87.5 ± 6.7	89.0 ± 9.3	1.50 ± 10.69	0.50	
Insulin (μIU/mL)						
PLA	27	8.4 ± 4.0	11.2 ± 9.6	2.7 ± 9.1	0.13	0.34
BHB	24	11.1 ± 6.7	10.0 ± 4.9	−1.2 ± 4.6	0.22	
Uric Acid (mg/dL)						
PLA	27	5.1 ± 1.4	5.2 ± 1.4	0.05 ± 0.86	0.76	0.77
BHB	24	5.2 ± 1.2	5.5 ± 1.3	0.28 ± 0.93	0.16	
Alkaline Phosphatase (U/L)						
PLA	27	67.7 ± 18.3	67.9 ± 19.7	0.19 ± 8.16	0.91	0.41
BHB	24	64.3 ± 19.5	63.9 ± 19.9	0.42 ± 7.23	0.78	
AST (U/L)						
PLA	27	20.4 ± 6.1	21.0 ± 5.7	0.63 ± 5.20	0.54	0.04
BHB	24	20.4 ± 6.6	19.3 ± 5.2	−1.08 ± 6.92	0.45	
ALT (U/L)						
PLA	27	20.0 ± 10.3	20.4 ± 9.3	0.41 ± 5.50	0.70	0.24
BHB	24	23.8 ± 15.6	19.0 ± 6.6	−4.75 ± 11.0	0.05	
Total Cholesterol (mg/dL)						
PLA	27	184.0 ± 32.4	184.3 ± 34.4	0.3 ± 21.0	0.95	0.24
BHB	24	191.8 ± 41.2	182.8 ± 37.9	−9.0 ± 19.5	0.03	
Triglycerides (mg/dL)						
PLA	27	98.9 ± 44.9	108.3 ± 42.0	9.37 ± 48.9	0.33	0.95
BHB	24	98.8 ± 42.9	111.9 ± 66.8	13.1 ± 52.9	0.24	
HDL Cholesterol (mg/dL)						
PLA	27	50.7 ± 11.9	51.1 ± 9.4	0.41 ± 7.0	0.77	0.07
BHB	24	52.7 ± 12.4	49.9 ± 12.5	−2.79 ± 3.7	0.001	
LDL Cholesterol (mg/dL)						
PLA	27	113.4 ± 27.5	111.5 ± 27.9	−1.89 ± 20.9	0.64	0.53
BHB	24	119.3 ± 33.9	110.5 ± 34.1	−8.79 ± 19.3	0.04	
Homeostatic Model Assessment of Insulin Resistance (HOMA−IR)						
PLA	27	1.89 ± 0.93	2.45 ± 2.05	0.56 ± 0.46	0.14	0.59
BHB	24	2.44 ± 1.59	2.24 ± 1.23	0.20 ± 0.40	0.42	
Total Cholesterol:HDL Ratio						
PLA	27	3.75 ± 0.80	3.65 ± 0.60	0.10 ± 0.20	0.38	0.85
BHB	24	3.74 ± 0.85	3.78 ± 0.82	0.04 ± 0.23	0.70	

* The between-group *p*-value was assessing the group × time interaction between week 0 and week 8.
